# Increased Activation Markers of Adaptive Immunity in Patients with Severe COVID-19

**DOI:** 10.3390/jcm13195664

**Published:** 2024-09-24

**Authors:** Abdulelah Aljuaid

**Affiliations:** Department of Clinical Laboratory Sciences, College of Applied Medical Sciences, Taif University, P.O. Box 11099, Taif 21944, Saudi Arabia; ab.aljuaid@tu.edu.sa or abdulelah.aljaied@gmail.com

**Keywords:** B and T cells, CD markers, adaptive immunity, SARS-CoV-2, COVID-19

## Abstract

**Introduction:** COVID-19 is a pandemic disease and is widespread over the world. This disease shows a 5.1% mortality. The understanding of the disease has expanded rapidly in many areas, including virological, epidemiological, clinical, and management dimensions. To better understand the inflammatory and immune profiles that impact the pathogenesis and development of severe COVID-19 symptoms, further studies are essential. This research aims to explore the inflammatory and adaptive immune responses associated with COVID-19, considering factors such as genetic diversity and environmental exposure among Saudi patients. The goal is to determine if patients with severe COVID-19 exhibit different disease phenotypes. **Materials and Methods:** This case-control study includes 115 participants (healthy and with COVID-19 infection), 55 of which had confirmed cases of COVID-19 in intensive care units (ICUs) at different hospitals in Makkah City, Saudi Arabia. Whole blood samples were collected from June to September 2021 for cellular analyses, and inflammation marker data were collected from hospital records. The expression of activation markers on B (CD27 and CD38) and T cells (CD27 and HLA-DR) was obtained using the flow cytometry technique. Also, serum was collected for cytokine measurements, including IL-6, INF-γ, and TNF- α. **Results:** The results indicated that lymphopenia and excessive T cell activation were more prevalent in severe cases than in healthy individuals. Furthermore, the results revealed that severe COVID-19 patients had an increased frequency of CD19+ B cells, with changes in B cell subsets. The current study implies impairment and changes in the phenotype of adaptive cells (including T and B cells), with an increase in HLA-DR molecules and inflammation markers with pro-inflammatory cytokines in severe COVID-19 cases. **Conclusions:** The current study implies impairment and changes in the phenotype of adaptive cells (including T and B cells), with an increase in HLA-DR molecules and inflammation markers in severe COVID-19 cases, which could be targeted for therapeutic interventions. This might be a valuable approach for the diagnosis and treatment of severe COVID-19 cases.

## 1. Introduction

Coronavirus disease 2019 (COVID-19) was first identified in China, and the virus has since spread worldwide. In March 2021, the World Health Organization (WHO) classified COVID-19 as a pandemic [1]. Most COVID-19 cases are asymptomatic or exhibit only mild symptoms, but some may develop severe or critical manifestations [2]. A common sign in COVID-19 patients is fever, with many also experiencing respiratory symptoms such as coughing, sore throat, dyspnea, and gastrointestinal symptoms like abdominal pain, vomiting, and diarrhea [3].

However, some patients may experience additional complications, such as acute respiratory distress syndrome (ARDS), accompanied by dyspnea and respiratory failure, necessitating mechanical ventilation to prevent fatalities [4,5]. Furthermore, severe COVID-19 cases can present cardiovascular complications, including cardiomyopathies, arrhythmias, and heart failure [4,6]. Research has shown an upregulation of cytokines and chemokines, such as IL-2, IL-6, IL-7, and TNF-α. These cytokines contribute to the cytokine storm observed in severe cases of both SARS and MERS, which can lead to dysregulation in the immune system, shock, organ failure, and potential mortality [7].

During COVID-19 infection, the host’s immune response initiates innate and adaptive immunity. Regarding the adaptive immune system’s participation, lymphocytes (B and T cells) play a critical role in defending against viral infection. It is well known that during viral infection, the human body requires a dominant Th1 response subset to control the disease, and cytokines and chemokines secreted by antigen-presenting cells (APCs) make naïve T cells differentiate to the Th1 subset. 

HLA DR markers are receptors found on the surface of cells, within histocompatibility complex (MHC) class II molecules. They play a role in presenting peptide antigens to the immune system, particularly to T helper cells, thus influencing the interaction between innate and adaptive immunity. These markers are predominantly present on antigen-presenting cells like macrophages, B cells, and dendritic cells. Various factors such as infection, inflammation, and immunosuppression can impact the expression of HLA DR markers. The reduced expression of these markers is linked to poor outcomes in ill patients, including a higher likelihood of secondary infections and mortality [8]. 

HLA DR markers are present on activated T cells, showing their role in the immune response. The amount of HLA DR expression on T cells can signify the level of T cell activation and inflammation in diseases like tuberculosis, hemophagocytic lymphohistiocytosis (HLH), sepsis, and multisystem inflammatory syndrome (MIS) [9,10]. Studies have shown that viral infection may induce changes in T cell markers, and one of these markers is HLA-DR. This marker is recognized as being associated with T cell activation [11] and is increased in the cytotoxic T cells (CTLs) of patients with viral infection. A study by Du et al. showed a correlation between the level of HLA-DR+ CD8+ in T cells and the severity of COVID-19 disease [12]. 

In parallel, the humoral immune response plays a vital role in releasing antibodies to fight against viral infection, which may help control diseases and prevent future encounters with the same viral infection [13]. B cells can be classified into different compartments in human peripheral blood. CD27 is a marker for identifying human memory B cells; studies have shown their rapid expression of CD27+ after stimulation, both in vitro and in vivo. In addition, CD38 is a surface molecule that functions as a signaling channel, leading to cellular activation, maturation, and proliferation [14]. Based on the expression of CD27 and CD38, B cell subsets can be identified as mature naive B cells (MN) (CD27−, CD38+), antibody-secreting plasmablasts (CD27+, CD38+), and resting memory B cells (RM) (CD27+, CD38−) [15]. T and B cells are implicated in the pathogenesis of acute and chronic viral infections [16]. Previous studies have shown that the COVID-19 disorder may impose immunological changes in T and B cells in infected patients [17].

Upon searching for research in Saudi Arabia related to studying the activity of the adaptive immune system, especially B and T cells, we found that the amount of research is minimal. Studies are rare and do not reflect how the body interacts with the coronavirus; moreover, most of the related studies are not based on laboratory experiments. Therefore, the scarcity of studies in the region encouraged the initiation of this exploratory study. Many studies have proven an increase in the activity of immune cells, but it should be considered that this activity may increase collator damage in the lungs. In addition, these immune cells might present a double-edged sword, as cells often co-express exhaustion markers like PD-1, hinting at a dysfunctional response that struggles to clear the virus effectively.

Therefore, in this study, we investigated and assessed the expression of CD27 and CD38 proteins on B cells to identify any changes in the circulating B cell subset in patients with severe COVID-19. We also examined the expression of activation markers (CD27 and HLA-DR) on CD4+ and CD8+ T cells to identify T cell states compared to normal individuals. The findings of this study may help increase our knowledge of immunological responses to COVID-19 infection and discover links between the presence of activated B and T cells and the clinical presentations of COVID-19 disease.

## 2. Materials and Methods 

### 2.1. Sample Collection and Processing

In this case-control study, COVID-19 patients were recruited from an isolation hospital in Makkah from June to September 2021. The inclusion criteria were all patients admitted to an ICU who were suffering from COVID-19 symptoms, and all cases were confirmed using PCR tests for COVID-19 on nasopharyngeal or throat swabs. These patients were admitted to the ICU because of severe complications of COVID-19 (such as fever, dyspnea, and respiratory failure). The patients’ average hospitalization was around 2–3 weeks. The inclusion criteria for healthy individuals were no history of COVID-19 infection based on the government record and no current flu symptoms. Participants who did not match the inclusion criteria were excluded from this study. Ethylenediaminetetraacetic acid (EDTA) whole blood samples were collected for cellular analyses. Inflammation markers, including C-reactive protein (C-RP), ferritin, and D-dimer data, were collected from hospital records. Procedures were performed after prior approval by the directorate of health affairs in Makkah City, Institutional Review Board (IRB) registration number HAP-02-T-067 (Approval Number: 427). This study was performed according to the principles of the Helsinki Declaration. 

### 2.2. Flow Cytometer Preparation and Staining 

We collected 2 of three ml of whole blood in an EDTA tube to isolate human peripheral blood mononuclear cells (PBMCs) and further characterize the target cells in the flow cytometer. We added the blood to one volume of 96%Ficoll-Paque (GE Healthcare, Little, Chalfont, Buckinghamshire, UK); then, we centrifuged the blood at 20.00 rpm for thirty minutes at 4 °C, and after centrifugation, the buffy coat was taken. After centrifugation, the sample was separated into 4 layers (including RBCS, Ficoll layer, Buffy coat, and Plasma). The PBMCs were removed from the thin interface between the upper plasma layer and the lower medium layer. Then, in a new FACS tube, the PBMCs were washed and centrifuged for 10–20 min at room temperature to remove any excess serum and the Ficoll. All samples were processed on the same day of collection.

**Figure 1:**
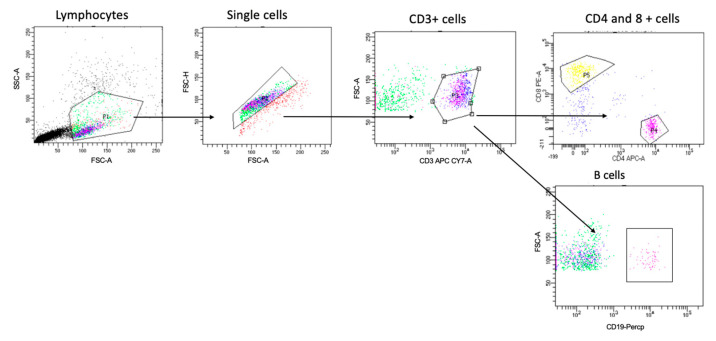
Gating strategy for identifying T and B cells. Cells were gated based on forward and side scatter, followed by single-cell and CD3+ cell gating. Cells were then gated for CD4+ cells, CD8 for T cells, and CD19 for B cells. Then, we gated further on three subsets of B cells (i.e., CD27+CD38+, CD27+CD38−, and CD27−CD38+) and HLA-DR expression on both CD4+ CD8+ T cell populations, as shown in Figures 4 and 5.

To study the PBMCs using the flow cytometer, all samples were washed with PBS before staining. To identify the different cells of interest in this study, the PBMCs were stained with antibodies for all the extracellular markers selected in Table 1. The gating strategy to identify the T cell and B cell populations is shown in Figure 1. Then, all samples were left for 30 min at 4 °C for the incubation period, protected from light, washed, and resuspended in FACS buffer. Since the multicolor flow cytometry was performed, with all runs, single stained samples were essential to determine the levels of compensation and prepare the machine to identify the positive and negative population from each color. This step prevented any spectral overlap between different fluorophores. Then, the samples were analyzed on the flow cytometer [5].

### 2.3. Cytokine Measurement 

Three proinflammatory cytokines were measured, including IL-6, IFN-γ, and TNF-α. Cytokine levels were quantified using high-sensitivity human enzyme-linked immunosorbent assay (ELISA) kits specific to each cytokine (MyBioSource, San Diego, CA, USA). Each serum sample was run in duplicate, following the manufacturer’s instructions

### 2.4. Statistical Analysis 

FlowJo software (Mac version 9) (Tree Star, Ashland, and Oregon) was used to analyze the flow data. Before each experiment, the machine was compensated, and a single stain was controlled for each antibody. To graph the data, GraphPad Prism 8 (Mac version 8.4.2) (La Jolla, CA, USA) and SPSS (Mac version 17.0) (IBM Corp., Armonk, NY, USA) were used to perform the statistical analysis. The Shapiro–Wilk normality test was performed to ensure the data were normally distributed. The parametric (unpaired T-tests) was used based on the normality test. If the *p*-value was <0.05, the differences in the result were considered statistically significant. 

## 3. Results

### 3.1. Demographic Characteristics of Study Participants 

In this study, 115 participants (healthy and with COVID-19 infection) were included. Overall, 55 had confirmed cases of COVID-19 and 60 were healthy individuals; the inclusion criteria for both were mentioned in the Materials and Methods. For all infected participants, the mean age was 51.89 ± 11.5. Gender diversity and some laboratory characteristics (including C-reactive protein (C-RP), ferritin, and D-dimer) are shown in Table 2. There were no significant differences in age (*p* ≤ 0.106), gender (male *p* ≤ 0.931 and female *p* ≤ 0.942), or smoking status (smoker *p* ≤ 0.890, non-smoker *p* ≤ 0.9521). 

**Figure 2:**
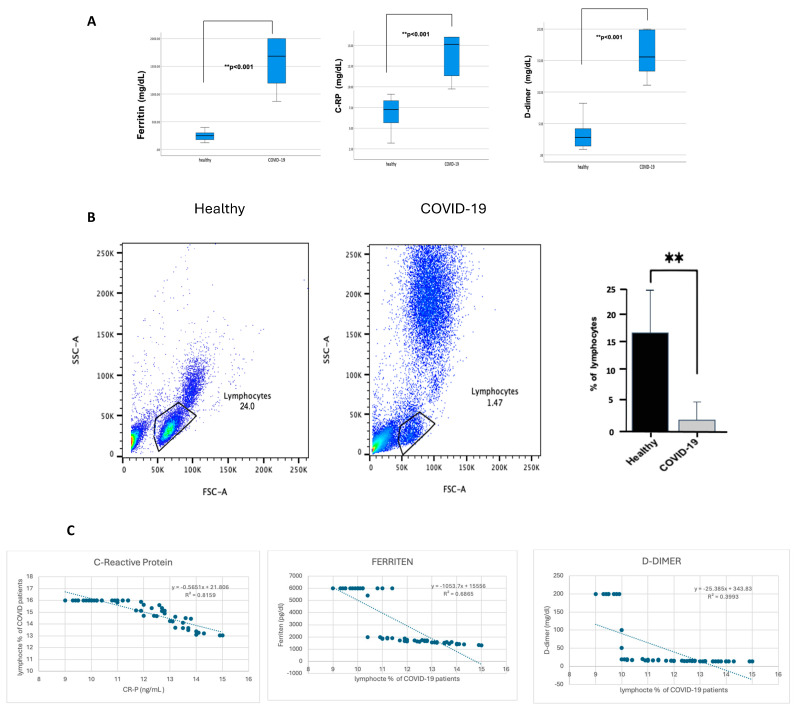
The blood of participants with COVID-19 showed a high percentage of CRP, ferritin, and D-dimer. In contrast, it showed a reduction in lymphocyte percentage, and CRP, ferritin, and D-dimer were negatively correlated with lymphocyte percentage reduction. The hospital lab analyzed the peripheral blood samples from either healthy controls or confirmed COVID-19 patients (infected). (**A**) Representative CRP, ferritin, and D-dimer boxplot for all healthy and COVID-19 participants. (**B**) Representative dot plots of the lymphocyte population using forward scatter (FSC), side scatter (SSC), and pooled data for lymphocytes among both healthy and COVID-19 patients, shown as percent of lymphocytes. (**C**) Correlation graph for CRP, ferritin, D-dimer, and the percentage of lymphocytes in COVID-19 patients. Unpaired T-tests were used to analyze the two groups, ** *p* < 0.001.

From this point, all further analyses were performed on patients with severe COVID-19 who showed a significant increase in inflammation markers. CRP, ferritin, and D-dimer were significantly higher (*p* ≤ 0.001) in patients with severe COVID-19 than in healthy individuals, as shown in Figure 2A. In contrast, lymphocyte percentage was considerably lower (*p* ≤ 0.001) in COVID-19 patients, as shown in Figure 2B. Of note, patients with severe COVID-19 indicated an increase in the granulocyte population compared with healthy individuals, but this statement requires further confirmation. To investigate whether lymphocyte depletion in patients with severe COVID-19 is associated with acute phase protein markers, the lymphocyte levels were correlated with CRP, ferritin, and D-dimer. Figure 2C demonstrates a strong negative correlation between CRP levels, ferritin, D-dimer, and the percentage of lymphocytes in COVID-19 patients, with a robust correlation coefficient (*p* < 0.00.1). However, the association with D-dimer is slightly weaker (*R*^2^ = 0.8159, 0.6865, and 0.3993, respectively).

**Figure 3:**
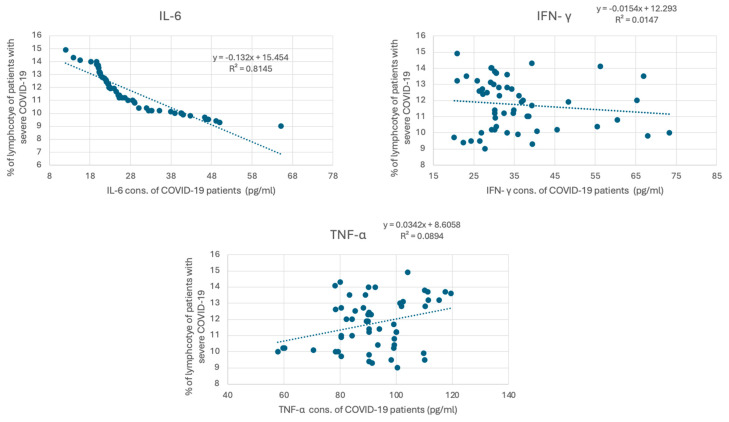
Correlation between cytokine concentration and the percentage of lymphocytes in COVID-19 patients. The graph presents the correlation between cytokine levels (pg/mL), including IL-6, IFN-γ, and TNF-α, in individuals diagnosed with severe COVID-19. IL-6 concentrations (pg/mL) versus percentage of lymphocytes. The trend line equation is y = −13.52x + 15.64 with an (R^2^) value of 0.8315. IFN-γ concentrations (pg/mL) versus percentage of lymphocytes. The trend line equation is y = −0.034x + 6.858 with an (R^2^) value of 0.0147. TNF-α concentrations (pg/mL) versus percentage of lymphocytes. The trend line equation is y = −0.029x + 8.658 with an (R^2^) value of 0.0894.

COVID-19 has been associated with the excessive production of proinflammatory cytokines, such as IL-6, IFN γ, and TNF-α. The measured cytokine levels are shown in Table 3. The mean concentration of IL-6 in healthy individuals was 6.87 ± 4.23 pg/mL, whereas, in patients with severe COVID-19, it was significantly elevated at 28.10 ± 10.80 pg/mL, with a *p*-value of <0.001. Similarly, INF-γ levels in healthy individuals averaged 4.65 ± 3.57 pg/mL, while in COVID-19 patients, they were markedly higher at 35.58 ± 12.40 pg/mL (*p* < 0.001). TNF-α levels also showed a significant increase, with healthy individuals having a mean of 54.53 ± 17.93 pg/mL and COVID-19 patients showing levels of 91.87 ± 13.83 pg/mL (*p* < 0.001). These findings underscore the elevated inflammatory response associated with COVID-19, highlighting the significant differences in cytokine profiles between the two groups. To investigate whether lymphocyte depletion in patients with severe COVID-19 is associated with cytokine levels, we correlated lymphocyte levels in patients with severe COVID-19 with their IL6, IFN γ, and TNF-α levels. Figure 3 demonstrates a strong negative correlation between IL-6 and the percentage of lymphocytes in COVID-19 patients, with a strong correlation coefficient (R^2^ = 0.814) (*p* < 0.001). However, the association between the percentage of lymphocytes in COVID-19 patients and IFN γ and TNF-α is weaker (R^2^ = 0.0147, and 0.0089, respectively).

### 3.2. COVID-19 Patients Displayed an Enhanced Frequency of CD19+ B Cells and an Increase in B Cell Subsets

**Figure 4:**
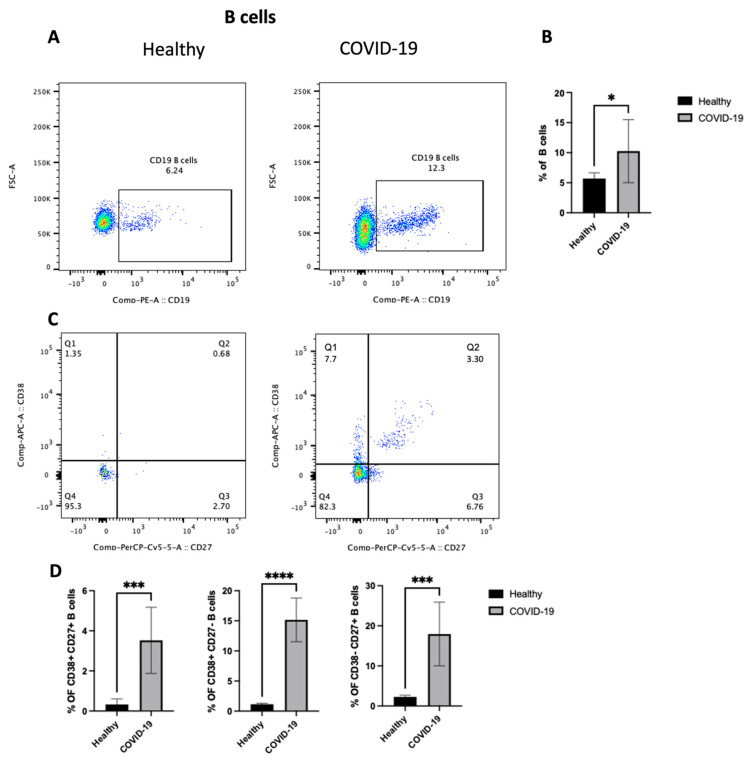
The PBMCs of patients with COVID-19 infection exhibit an enhanced frequency of CD19+ B cells and an increase in three subsets of B cells (i.e., CD27+CD38+, CD27+CD38−, and CD27−CD38+). Peripheral blood samples were collected from either healthy controls or ICU-confirmed COVID-19 patients. PBMCs were isolated from whole blood using Ficoll from healthy and COVID-19 patients and analyzed by flow cytometry. CD19+ B cells were divided into three subsets based on the expression of CD27 and/or CD38. (**A**) A dot plot showing the B cell population after gating on singlet, CD3−, and CD19+ populations from both the infected and health groups. (**B**) A representative graph for the percentage of total B cells between healthy and COVID-19 patients. (**C**) Representative dot plots of the B cell population after gating on singlet, CD3−, and CD19+ populations. Then, gating on either CD27 vs. CD38 expression on B cells for both healthy and patient samples. (**D**) Data for the percentage of CD27+CD38+, CD27+CD38−, and CD27-CD38+ on B cells in healthy and COVID-19 patients, shown as a percent of total B cells. Data represent two independent experiments and are shown as mean ± SD. Unpaired T-tests were used to analyze the two groups. * *p* < 0.05, *** *p* < 0.0001, **** *p* < 0.00001.

To identify the potential mechanism(s) by which the COVID-19 virus drives inflammation in the lung, the changes in the adaptive immune cells (focusing on B cells) in the PBMCs from patients who were confirmed to be positive for COVID-19 after PCR testing was studied. First, as shown in Figure 4A,B, COVID-19 patients showed an increase in the total B cell population (*p* < 0.05) compared with healthy individuals. However, they had lymphocytopenia, as shown in Figure 2B. Then, as we mentioned previously, B cells were classified into different compartments in human peripheral blood based on the expression of CD27 and CD38. So, the next step was to explore any changes in the three subsets of B cells, including CD27+CD38+, CD27+CD38−, and CD27-CD38+. The data highlight an increase in the frequency of all three subsets (*p* < 0.0001) (Figure 4C,D), mature naive B cells (MN) (CD27−, CD38+), antibody-secreting plasmablasts (CD27+, CD38+), and resting memory B cells (RM) (CD27+, CD38−). Thus, these results indicate an increase in the number of activated B lymphocytes in peripheral blood after COVID-19 infection. 

### 3.3. The PBMCs of Patients with Severe COVID-19 Indicate Increased HLA-DR on CD4+ and CD8+ T Cells

**Figure 5:**
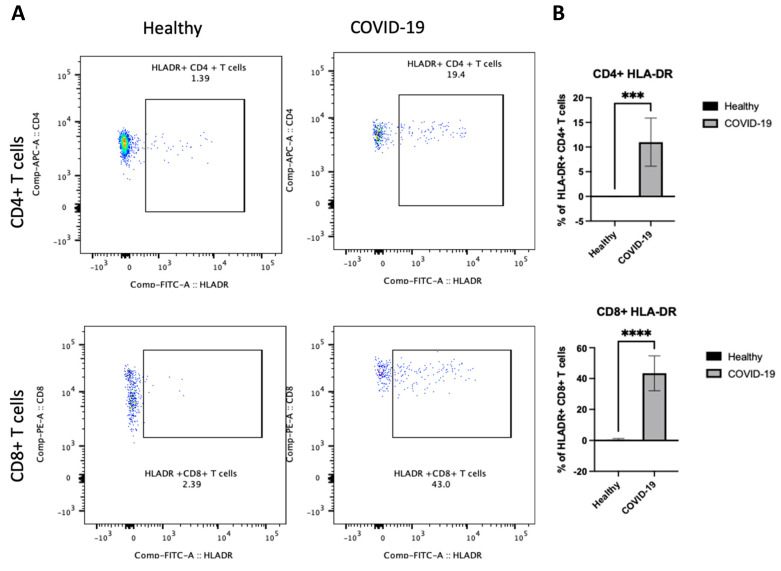
The PBMCs of patients with COVID-19 exhibit an enhanced frequency of HLA-DR on CD4+ and CD8+ T cells. Peripheral blood samples were collected from healthy controls and ICU-confirmed COVID-19 patients (infected). PBMCs were isolated from whole blood using Ficoll from healthy and COVID-19 patients and analyzed using flow cytometry; HLA-DR expression was identified on CD4+ and CD8+ T cells. (**A**) Representative dot plots of HLA-DR expression on CD4+ CD8+ T cell populations after gating on singlet, CD3+, CD4+, and CD8+ populations. (**B**) Pooled data for the percentage of HLA-DR expression on CD4+ and CD8+ T cells among healthy and COVID-19 patients, shown as a percent of total T cells. Data are shown as mean ± SD. We used an unpaired T-test to analyze the two groups. *** *p* < 0.0001, ****; *p* < 0.00001.

After demonstrating differences in B cells in PBMCs following COVID-19 infection, the next step sought to determine whether there were any changes in the other arm of cellular immunity and examined the T cells. We were interested in investigating whether the frequency of HLA-DR on both CD4+ and CD8+ T cells was altered and whether the activity states of T cells, as these cells have previously been associated with disease severity [10]. We first assessed the activation status of T cells by examining HLA-DR. The data showed that COVID-19 patients had a high frequency of HLA-DR expressing CD4+ and CD8+ T cells (*p* < 0.00001), as shown in Figure 5A,B. Thus, these results indicate that an active T cell immune response occurs in peripheral blood after COVID-19 infection. 

## 4. Discussion 

The current study evaluates the immunophenotyping of adaptive immunity, including T and B cells, among patients with severe COVID-19 and studies the association between adaptive activation statutes and clinical severity in COVID-19 patients. Although many studies around the world investigated the change in adaptive immunity to severe COVID-19, this study was conducted in Saudi Arabia to determine if factors like genetic diversity, environmental exposure, and viral variants show different disease phenotypes in severe COVID-19. 

The data showed that all COVID-19 confirmed cases had increased inflammation markers, such as C-RP, D-dimers, and ferritin [18]. This is consistent with many studies and reviews that have indicated poor outcomes in COVID-19 patients related to elevated serum levels of CRP, PCT, D-dimer, and ferritin. The literature shows, along with other biomarkers included in this study, an increase in inflammatory cytokine expressions, such as IFN-γ, IL-6, and TNF-α [15]. 

Thus, this hyperinflammation status may lead to cardiopulmonary collapse and multi-organ failure, as seen in many patients with severe COVID-19. Moreover, the data indicated that patients with severe COVID-19 showed an increase in the granulocyte population, as noted in the side and forward scatter, compared with healthy individuals. However, this statement requires further confirmation, as mentioned before. It is well known that granulocytes are effector cells that predominate during the early or acute phase of the innate immune response. Therefore, they were not expected to be observed because of their short life and because patients suffered from the disease for more than two weeks. 

The data showed a reduction in the percentage of lymphocytes in the peripheral blood of COVID-19 patients, which is consistent with the literature [3,19,20]. Furthermore, this study revealed a negative correlation between CRP, ferritin, D-dimer, and the percentage of lymphocytes in COVID-19 patients, which is consistent with existing literature. This suggests that acute-phase protein levels rise while lymphocyte percentages tend to decrease. Previous research has demonstrated that the ratio of acute-phase protein levels to lymphocytes can serve as a significant predictor of adverse outcomes in COVID-19 patients [2,21]. One reason to explain this phenomenon is that acute-phase proteins are part of the innate immune response, while lymphocytes play a crucial role in adaptive immunity. Dysregulated immune responses like increasing the level of acute phase proteins may lead to lymphocyte depletion and worsen disease outcomes. 

The data presented in Table 1 align with previous studies reporting elevated cytokine levels in COVID-19 patients compared with healthy controls. Elevated IL-6 levels have been consistently associated with severe disease and cytokine storm phenomena in COVID-19 patients [22,23]. Similarly, increased INF-γ and TNF-α levels in COVID-19 patients corroborate findings from other research indicating heightened immune activation and inflammation in response to SARS-CoV-2 infection [24,25]. 

Also, the data indicated a strong correlation between IL-6 levels and reduced lymphocyte percentage in COVID-19 patients, which supports findings from other studies highlighting IL-6’s role in driving immune dysregulation. Elevated IL-6 is known to be a key player in the cytokine storm associated with severe COVID-19, which can lead to lymphopenia and impaired immune function [2,26]. Conversely, the less clear correlations between INF-γ and TNF-alpha and the lymphocyte percentage suggest that these cytokines might not directly influence lymphocyte counts in the same way. While INF-γ and TNF-alpha are critical in the inflammatory response and can affect immune cell functions, their effects on lymphocyte levels may be more complex or influenced by additional factors not fully captured in this study. These findings align with some studies indicating that while INF-γ and TNF-alpha are elevated in severe COVID-19, their relationship with the lymphocyte count may be less direct [27,28]. Further research is needed to clarify the mechanisms by which these cytokines interact with lymphocyte dynamics in the context of COVID-19. 

However, the data indicated that patients with COVID-19 infection developed potent adaptive immunity, as CD4 and CD8 T cells demonstrated elevated levels of HLA-DR+ [12]. The reason why patients with severe COVID-19 develop lymphopenia requires further investigation, especially since studies have shown that lymphocytes do not express a high level of angiotensin-converting enzyme 2 (ACE2), the cell entry receptor for the coronavirus [27,28]. Other studies have indicated no virus detection in the peripheral blood of COVID-19 patients [3,29]. Therefore, it suggested that the lymphopenia in COVID-19 patients was not due to direct infection of the cell. 

The results showed an increase in B cells of all subtypes and a decrease in T cells despite the increase in active T cells. This may be because the immune system is in a state of alert to increase the activation of T cells and their absence in the blood or that most of the T cells went to the site of infection to fight the active condition of the virus and prevent its spread in the alveolar epithelium. The data indicated that CD4+ T cells showed expression of HLA-DR in peripheral blood. A study by Tippalagma et al. indicated that patients with bacterial pulmonary infection showed higher antigen-specific T cell activation and inflammation, specifically for HLA-DR molecules in patients with bacterial infection compared to healthy individuals. However, the same study showed that these effector HLA-DR+ CD4 T cells displayed different characteristics like an expression of effector memory phenotype (CD45R-CCR7-) and increased the expression of cytotoxic molecules and proinflammatory cytokines like TNF-α. Other works also demonstrated that memory CD4+ T cells could rapidly migrate to the tissue to provide antigen elimination. However, we did not investigate the further characterization of HLA-DR CD+4 T cells to determine if they are memory T cells in the periphery or not [10]. A study by Song JW et al. revealed no significant difference in HLA-DR+ CD4+ T cells between patients with severe COVID-19 and healthy individuals. However, they observed an increase in HLA-DR in the CD4+CD38+ T cell population, which they did not focus on [28].

Consistent with this study’s result, a study by Song, JW et al. showed a significant increase in HLA-DR expression in CD8+ T cells in patients with severe COVID-19 compared with healthy individuals. Although the data showed a reduction in lymphocyte frequencies, CD8 T cells in patients with severe COVID-19 indicated an over-activation of T cells, similar to other acute infections such as malaria. Therefore, this study and song JW et al.’s study imply that severe patients who persist in the disease a long time after illness show an increase in activated CD8+ T cells (defined by HLA-DR+), which highlights their role in immunopathogenesis in severe COVID-19 cases. We suggest that using lymphocyte subset cell counts with an HLA-DR marker could be beneficial in predicting the outcome of COVID-19 infections [28]. 

The COVID-19 patients in this study displayed significant differences in CD19+ peripheral blood B cell frequency. The data also indicated a higher percentage of mature naive B cells (MN) (CD27−, CD38+), antibody-secreting plasmablasts (CD27+, CD38+), and resting memory B cells (RM) (CD27+, CD38−) in COVID-19 patients compared with healthy individuals. This was also observed in Sosa-Hernández et al.’s work [14]. Regarding the antibody-secreting plasmablast subset, the reason for their increase in COVID-19 remains unclear; however, several works indicated that an early rise in antibody-secreting plasmablasts was essential to produce high levels of SARS-CoV-2-specific antibodies [14,29]. In other viral infection scenarios, patients with dengue virus infection showed increases in the antibody-secreting plasmablast subset, which was correlated with the severity of the disease. The data indicated an alteration in B cell subsets in the peripheral blood of COVID-19 patients. 

This study has limitations like sample size, sex incompatibility, and variety of participant ages, so more investigation is needed to link the observations with the severity of COVID-19. More importantly, this study showed a lack of functional data, and it was difficult to determine the precise functional characteristics of any cells that showed an increase in their population for patients with severe COVID-19. However, this study could be more helpful if it was conducted on a larger population and included different activation markers that could support the treatment and diagnosis in patients with severe COVID-19.

## 5. Conclusions

The immune system is quite complex, and understanding how its response, which demonstrates a strong specificity, toward a pathogen holds significant importance. Understanding this aspect aids in determining the best vaccine strategies that can trigger enduring immunity against COVID-19. Furthermore, exploring immunity across populations is crucial as it sheds light on how genetic, environmental, and historical elements influence the body’s response to SARS-CoV-2. Varied populations may exhibit levels of susceptibility or resilience to infections, leading to outcomes and complications. Additionally, this research plays a role in monitoring the appearance and transmission of strains of SARS-CoV-2 that might evade immune defenses, potentially resulting in reinfections or breakthrough cases. In conclusion, the current study indicates changes in the phenotype of adaptive cells (including T and B cells) and increased inflammation markers in severe COVID-19 cases. It will help to understand the adaptive immune response to the coronavirus, which might help diagnose and treat patients with severe COVID-19. 

## Figures and Tables

**Table 1:** All antibodies that were used and their fluorochrome and concentrations.

Antibody Target	Fluorochrome	Concentration Used
CD4 and CD38	APC	1 μg/mL
CD19	PerCP	1 μg/mL
CD8	PE	1 μg/mL
CD27	PerCP Cy5.5	1 μg/mL
HLA-DR	FITC	1 μg/mL
CD3	APC Cy7	1 μg/mL

Cluster of Differentiation (CD), Human Leukocyte Antigen-DR isotype (HLA-DR), Allophycocyanin (APC), peridinin–chlorophyll–protein complex (PerCP), Phycoerythrin (PE), Peridinin Chlorophyll Protein Complex–Cyanine (PerCP Cy5.5), fluorescein isothiocyanate (FITC), and Allophycocyanin Cyanine (APC Cy7). Microgram per milliliter (μg/mL).

**Table 2:** Demographic and laboratory characteristics of the study participants.

Parameters	COVID-19 Participant (n = 55)	Healthy (n = 60)	*p*-Value
Age (years) (mean ± SD)	51.89 ± 11.5	40.06 ± 18.133	0.106
Gender Female (n, %) Male (n, %)	22 (40%) 33 (60%)	24 (40%) 36 (60%)	0.931 0.942
Smoker Non-smoker	9 (16.3%) 46 (83.4%)	9 (15%) 51 (85%)	0.890 0.952
CRP (mg/dL) (mean ± SD)	15.14 ± 1.06	5.60 ± 2.56	<0.001
Ferritin (mg/dL) (mean ± SD)	1739.37 ± 638.77	337.11 ± 244.77	<0.001
D-dimer (ng/mL) (mean ± SD)	18.10 ± 13.25	3.63 ± 2.33	<0.001

Data are mean ± standard division (SD), number, and (%); unpaired test for age, C-reactive protein CRP, ferritin, and D-dimer; Chi-square test for gender and smoker.

**Table 3:** Comparative IL-6, INF-γ, and TNF-α levels in healthy individuals and COVID-19 patients.

Parameters	Healthy (Mean±) (pg/mL)	COVID-19 (Mean±) (pg/mL)	*p*-Value
IL-6	6.87 ± 4.23	28.10 ± 10.80	<0.0001 ***
INF-γ	4.65 ± 3.57	35.58 ± 12.40	<0.0001 ***
TNF-α	54.53 ± 17.93	91.87 ± 13.83	<0.0001 ***

Data are as mean ± standard division (SD), number, unpaired test for IL-6, INF-γ, and TNF-α, *** *p* < 0.0001.

## Data Availability

Data are available on request.
